# Pregnancy effect on disease activity in women with multiple sclerosis treated with cladribine

**DOI:** 10.1007/s00415-024-12291-7

**Published:** 2024-04-03

**Authors:** E. Signoriello, M. Foschi, R. Lanzillo, J. Frau, E. Cocco, G. Borriello, A. Ianniello, M. Trotta, D. Landi, G. T. Maniscalco, F. Ruscica, S. Toscano, F. Patti, A. Zanghì, E. D’Amico, R. Fantozzi, D. Centonze, G. Lus, S. Bonavita

**Affiliations:** 1 Multiple Sclerosis Center, Second Division of Neurology, Department of Advanced Medical and Surgical Sciences, University of Campania Luigi Vanvitelli, Naples, Italy; 2 Department of Neuroscience, MS Center—Neurology Unit, S. Maria Delle Croci Hospital, AUSL Romagna, Ravenna, Italy; 3 Department of Biotechnological and Applied Clinical Sciences, University of L’Aquila, L’Aquila, Italy; 4 Department of Neurosciences, Reproductive and Odontostomatological Sciences, Federico II University, Naples, Italy; 5 Multiple Sclerosis Center, Department of Medical Sciences and Public Health University of Cagliari, Binaghi Hospital Cagliari/Italy, Cagliari, Italy; 6 San Pietro Fatebenefratelli-Hospital-MS Center Rome, Rome, Italy; 7 Department of Human Neuroscience, Sapienza University of Rome, Rome, Italy; 8 Unit of Neurology A.O. Annunziata, Cosenza, Italy; 9 Multiple Sclerosis Clinical and Research Unit, Department of Systems Medicine, University of Rome Tor Vergata, Tor VergataUniveristy Hospital, Rome, Italy; 10 Neurological Clinic and Multiple Sclerosis Center, A Cardarelli Hospital, Naples, Italy; 11 U.O.C. Neurologia E Centro SMFondazione Istituto G. Giglio, Cefalù, PA Italy; 12 Department “GF Ingrassia”, Section of Neurosciences, University of Catania, Catania, Italy; 13 Department of Medical and Surgical Sciences, University of Foggia, Foggia, Italy; 14 Unit of Neurology, IRCCS Neuromed, Pozzilli, IS Italy

**Keywords:** Cladribine, Multiple sclerosis, Pregnancy, Breastfeeding, Disease activity

## Abstract

**Introduction:**

**C**ladribine is an oral immune reconstitution therapy for relapsing multiple sclerosis (RMS). Hormonal and immune changes are responsible for the decline of disease activity in the third trimester of pregnancy and disease reactivation in the early post-partum period.We investigate the impact of pregnancy on disease activity in women with MS who conceived after cladribine treatment.

**Methods:**

We recruited women of childbearing age with relapsing–remitting MS (RRMS) who became pregnant or not after being treated with cladribine. For both groups, demographic, clinical and radiological data were collected 1 year before and after treatment during a mean follow-up of 3.53 years. We compared disease activity over time between groups using variance analysis for repeated measures.

**Results:**

48 childbearing women were included. 25 women had a pregnancy after a mean of 1.75 years from the first treatment cycle. Women with or without pregnancy did not differ in demographics or pre-cladribine disease activity. No significant differences in disease activity or EDSS worsening were found between women with or without pregnancy.

**Discussion:**

Our findings suggest that pregnancy does not appear to influence disease activity and disability in women previously treated with cladribine; further studies with larger numbers and longer follow-up are needed to confirm this finding.

## Introduction

Multiple sclerosis (MS) is an autoimmune and neurodegenerative disorder of the central nervous system (CNS) characterized by a young age at onset and a female sex predominance [1]. Hormonal and immune changes occurring during pregnancy may significantly impact MS activity [2]. It is recognized that the risk of relapse declines during pregnancy and rebounds in the early postpartum period, eventually returning to a level comparable to the pre-pregnancy state within the 1st year postpartum [3]. Consequently, patients in childbearing age are often counseled already at the time of MS diagnosis to plan future pregnancies. This planning often plays a pivotal role in guiding the selection of appropriate disease-modifying therapies (DMTs). Indeed, few DMTs, such as injectables, can be continued until conception or even during pregnancy, while others might require a specific washout period before attempting conception due to their potential fetal toxicity [4].

Cladribine, administered as an oral pulsed therapy, involves a cumulative dose of 3.5 mg/kg over 2 years, with one annual course lasting up to 10 treatment days. Acting as a prodrug, cladribine enters cells and, once activated, disrupts DNA synthesis, leading to selective apoptosis of lymphocytes [5]. MS activity control typically persists for approximately 4 years after cladribine initiation [6].

Reproductive toxicology studies on animals showed no adverse effects on female and male fertility or offspring development [7]. However, intravenous cladribine wasshown was teratogenic in mice and rabbits [7]. Given the potential teratogenicity at higher dose in animals, caution is required for conception during and after cladribine administration; indeed, to avoid the risk of exposure for the conceptus, a 6-month period of contraception is recommended after the intake of the last cladribine tablet [8]. Observations from the Integrated Analysis of Safety during the Clinical Development Program of cladribine were generally consistent with epidemiological data on pregnancy outcomes for the general population or women with MS. Specifically, there were no differences in the rate of congenital malformations between those exposed to cladribine treatment or within 6 months after the last dose [6] and those administered placebo. Anyway, there is currently a lack of information regarding the effect of pregnancy on disease activity in women with MS who conceived after the first or 2nd year of cladribine treatment.

In this retrospective study, we compared measures of MS activity in women of childbearing age treated with cladribine between (1) patients who conceived or not; (2) patients with pregnancy occurring between the first and second cycle or after 2 years of treatment; (3) patients who breastfed or not. Additionally, we evaluated pre- and post-pregnancy disease activity and collected safety data.

## Materials and methods

We retrospectively recruited from 12 Italian MS centers, all RR-MS women of childbearing age treated with cladribine tablet in the clinic and with regular follow-up visits. After study enrollment, women with not yet complete follow-up to meet the inclusion criteria (i.e., ongoing pregnancy, too short [post-pregnancy] follow-up, etc.…) were prospectively followed up at each participating center. The study was approved by the local ethics committees and all participants signed an informed consent. Inclusion criteria were: (i) female patient with RR-MS treated with cladribine in the clinic; (ii) patients who became or not pregnant after a treatment cycle (either the first or the second). Exclusion criterion was the use of other DMTs during pregnancy or after delivery. From the patients’ data records, we collected the following demographic and clinical information for each patient: year of birth; year of diagnosis; disease duration, number of treatments before cladribine, last DMT before cladribine start, disease activity in the year before cladribine defined as annualized relapse rate (ARR), and MRI activity (new T2 lesions and number of gadolinium enhancing lesions in the last MRI practiced within 6 months prior to cladribine start); disease activity after cladribine start defined as the number of relapses, date of occurred relapse, number of new T2 lesions or gadolinium enhancing lesions accumulated after cladribine therapy (either before or after pregnancy). MRI was performed after 6 months of cladribine start and subsequently every year after treatment. We also collected the lymphocytes count at baseline, after 3 months, 1 year and 2 years of treatment, and the expanded disability status scale (EDSS) score at cladribine start and after treatment (every year and at last follow-up), the date of EDSS worsening and EDSS at last follow-up. EDSS worsening was considered if patients presented an increase in EDSS during the follow-up period (either ≥ 1.5 points for patients with a baseline EDSS of 0, ≥ 1.0 point for patients with a baseline EDSS of 1–5 and by 0.5 points for patients with a baseline EDSS of ≥ 5.5 [9]).

We collected the following pregnancy information: date of last menstruation, date of delivery, data on breastfeeding and safety data about the newborn, (length and weight at birth and any malformation). In the first part of the study, we divided the whole cohort into two groups: patients with (P) or without pregnancy (NP) after the cladribine administration to compare disease activity and disability progression after cladribine treatment between the two groups. In the second part of the study, we evaluated pre- and post-pregnancy disease activity (ARR, MRI activity and EDSS worsening) in the P group to investigate the possible reactivation of disease after delivery.

Then we divided the P group into P-early or P-late if the pregnancy occurred, respectively, between the first or second cycle or after the second cycle of treatment to compare disease activity between these two groups; lastly, the P group was divided into subgroups according to breastfeeding.

### Statistical analysis

Demographic and clinical parameters have been reported as mean and standard deviation or as percentage and frequency for continuous or categorical variables. Baseline demographic and clinical characteristics of P and NP patients were compared with parametric and non-parametric *U* Mann–Whitney test for independent sample and Pearson Chi squared test was used for categorical variables.

Longitudinal comparisons of ARR, EDSS, lymphocytes counts and measures of MRI activity between P and NP patients were performed using variance analysis for repeated measures.

The same was performed for patients with P-early or P-late and with or without breastfeeding.

In a descriptive way, we compared disease activity and EDSS pre- and post-pregnancy with a Student's t test for paired measures.

The statistical significance level was set at a p value of < 0.05.

## Results

### Disease outcome comparison between patients with or without pregnancy (P vs NP)

We recruited 48 RR-MS women of childbearing age, treated as in the clinic with cladribine tablets, mean age 34.3 years (years) (sd 6.12), with a mean disease duration of 7.04 years (sd 3.43) and mean treatment follow-up 3.53 years (sd 1.64). Twelve patients were naïve to treatment before cladribine therapy, and 34 patients were switchers to cladribine. Of these, 54.3% switched from a mild–moderate efficacy (IFNs, GA, dimethyl fumarate, teriflunomide) and 19.6% from high efficacy treatments (fingolimod, natalizumab). Seven switched patients were P women and five switched patients were NP women, with no prevalent difference between the two groups; at baseline, switched patients had higher disease duration (7.79 ± 3.08 years vs 4.91 ± 3.62 years *p* = 0.02), lower number of gd + lesions (0.63 ± 0.78 vs 1.8 ± 1.5 *p* = 0.02) and higher EDSS (1.8 ± 0.95 vs 0.8 ± 0.6 *p* = 0.003). After the first or second cycle of cladribine, 25 women had a full-term pregnancy; 2 women had a spontaneous abortion, one at 8 weeks (after 8 months from the first cycle of cladribine) and the other at 7 weeks (after 27 months from the first cycle of cladribine). In these two patients, we did not observe any clinical or MRI reactivation after abortion. Demographic and clinical characteristics of P (*N* = 25) and NP patients (*N* = 21) are reported in Table 1; the two groups did not differ at baseline in terms of mean age, disease duration, pre-cladribine disease activity, disability as measured by EDSS and number of DMTs before cladribine treatment. Table 2 displays the clinical characteristics of P patients and newborns; mean time between the first cycle of cladribine and last menstrual period (LMP) was 1.75 years (sd 0.85); we divided the groups into P patients who conceived between the first and second cycle (P-early) and patients who conceived after the second cycle (P-late); we registered five P-early patients who had a mean interval between the first cladribine cycle and LMP of 6.1 months (sd 2.8), median interval 6,9 months (range 0.5–9 months). We had a patient exposed to cladribine after 15 days from the last LMP. For the 20 P-late patients who conceived after the second cycle, the mean interval from the II cycle to LMP was 1.09 years (sd 0.84) and the median interval was 9 months (range 2–51 months).

**Table 1:** Demographic and clinical data at baseline in patients with (*P*) and without pregnancy (NP)

	*P* = 25	NP = 21	*p*
Mean age (ys; sd)	34.98 ± 4.34 (27–43)	33.57 ± 4.34 (23–46)	0.24
Mean disease duration (ys;sd)	7.25 ± 3.41 (3.2–13.7)	6.78 ± 3.52 (2.4–13)	0.51
Mean number of treatments before (ys; sd)	1.1 ± 0.97 (0–4)	1.0 ± 0.77 (0–4)	0.71
Mean number new T2 lesions last MRI before clad (ys;sd)	1.56 ± 2.06 (0–10)	1.62 ± 1.89 (0–6)	0.94
Mean number gd + lesions last MRI before clad (ys; sd)	0.76 ± 0.83 (0–3)	1.2 ± 1.47 (0–5)	0.38
Mean follow up after cladribine start (ys; sd)	3.84 ± 2.07 (2.1–13)	3.17 ± 0.80 (2.2–5)	0.1
Mean EDSS at clad start (ys;sd)	1.39 ± 1.03 (0–4)	1.75 ± 0.85 (0–3.5)	0.28
Mean number of relapses previous year before clad (ys; sd)	1.08 ± 0.75 (0–3)	0.85 ± 0.65 (0–2)	0.33

**Table 2:** Clinical characteristics of P patients and newborns

*P*	
Mean time between I clad cycle and data of last LMP (ys; sd)	1.75 ± 0.85
Mean infant weight (gr; sd)	3117 ± 458.9
Mean infant length (cm; sd)	49.4 ± 1.7
% of breastfeeding P	64% (16/25)
Mean time of exclusive breastfeeding (months; sd)	4 ± 2.4

The P patients had 11 cesarean and 14 natural deliveries without complications in the mother; all newborns were healthy at birth except for a case with cardiac malformation. Specifically, we recorded a newborn with patent foramen ovale (PFO) from a P patient with 12 months interval between the last cladribine tablet and LMP. Most (64%) of the P patients breastfed (16/25) for a mean time of 4 months (sd 2.4).

**Fig. 1:**
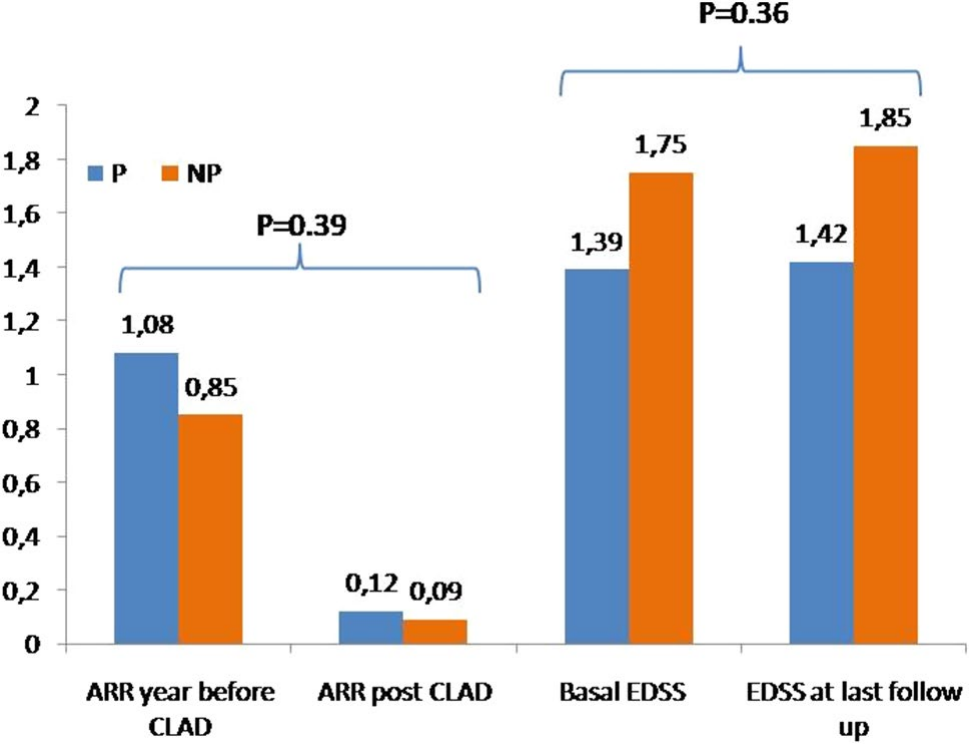
Mean ARR and EDSS before and after starting cladribine treatment in patients with (P) and without (NP) pregnancy

Regarding disease activity, we registered a significant reduction of ARR after cladribine treatment in both groups (P and NP), respectively, from 1.08 (sd 0.75) in the year before cladribine therapy to 0.12 (sd 0.06) after cladribine treatment for the P group (*p* = 0,001) and from 0.85 (sd 0.65) in the year before to 0.09 (sd 0.30) after cladribine therapy for the NP group (*p* = 0.001). No differences were documented over time and between groups (*p* = 0.39) (Fig. 1).

For disability progression, we observed an EDSS stability, in particular for the P group. EDSS at baseline was 1.33 (sd 1.11) and EDSS at last follow-up was 1.36 (sd 0.89) (*p* = 0.83), while the NP group registered a mean EDSS at baseline of 1.73 (sd 0.88) and EDSS at the last follow-up of 1.6 (sd 0.91) without significant differences (*p* = 0.16) between groups. Moreover, for the EDSS we registered nosignificant differences over time and between group P vs NP (*p* = 0.36) (Fig. 1). We analyzed disability progression in patients that did not present relapses to detect PIRA (progression independent of relapse activity), and of these only one patient in the P group presented with PIRA. For the other patients, an EDSS worsening was associated with relapse activity (RAW).

**Fig. 2:**
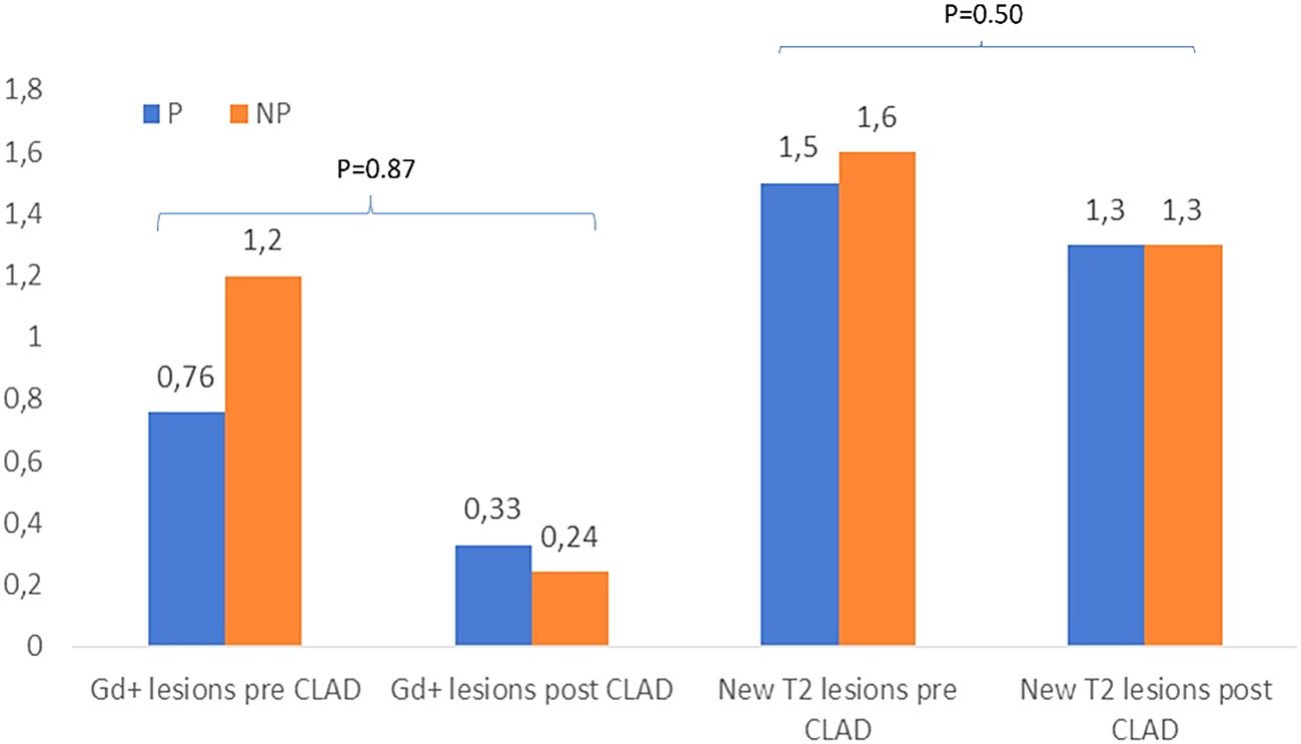
Mean new T2 lesions and gd+ lesions before and after starting cladribine treatment in patients with (P) and without (NP) pregnancy

As regards MRI measures of disease activity, we observed a post-cladribrine reduction of the mean number of new T2 lesions from 1.5 (sd 2.1) to 1.3 (sd 2.9) within the P group and from 1.6 (sd 1.8) to 1.3 (sd 1.9) within the NP group. There was also a reduction in the mean number of gd + lesions before and post-cladribine, respectively, from 0.76 (sd 0.83) to 0.33 (sd 0.86) for the P group and from 1.2 (sd 1.4) to 0.24 (sd 0.70) for the NP group. We did not find significant differences over time between the P and NP group (p = 0.87 for new T2 lesions and 0.50 for gd + lesions) (Fig. 2).

The percentage of treatment switch after cladribine was 16.6% (8/48 patients), with no significant differences between the P and NP group; in fact, 5 P patients and 4 NP patients switched to other treatments. In the whole cohort, mean time to treatment switch was 2.59 years ± 1.5; in particular, after 2.36 years ± 1.16 in P patients and 2.99 years ± 0.48 in NP patients (*p* = 0.33).

### Disease outcome comparison pre- and post-pregnancy

Among the 23 patients who had pregnancy, we found a statistically significant reduction in the ARR pre- versus post-pregnancy (1.08 sd 0.75 vs 0.12 sd 0.33 *p* = 0.001), a reduction of gd + lesions at MRI pre- versus post-pregnancy (0.79 sd 0.83 vs 0.33 sd 0.86 *p* = 0.05) and no differences between the mean EDSS pre-cladribrine and at last follow-up (1.35 sd 1.05 vs 1.3 sd 0.84; *p* = 1.0).

### Disease outcome comparison between patients with early or late pregnancy (P-early vs P-late)

The P-early and P-late groups did not differ in terms of age, disease duration, number of DMTs and disease activity (relapse, MRI and EDSS worsening) before cladribine initiation (Table 3).

**Table 3:** Demographic and clinical data at baseline in patients with *P*-early or *P*-late

	*P*-early = 5	*P*-late = 20	*p*
Mean age (ys; sd)	36.2 ± 4.1	34.8 ± 4.5	0.53
Mean disease duration (ys;sd)	8.34 ± 4.09	7.1 ± 3.3	0.33
Mean number of treatments before (ys; sd)	2 ± 1.4	0.9 ± 0.7	0.75
Mean number new T2 lesions last MRI before clad (ys;sd)	1.2 ± 0.95	1.7 ± 2.2	0.96
Mean number gd + lesions last MRI before clad (ys; sd)	0.4 ± 0.54	0.89 ± 0.87	0.29
Mean EDSS at clad start (ys;sd)	2.2 ± 1.5	1.2 ± 0.72	0.29
Mean number of relapses previous year before clad (ys; sd)	1. ± 0.70	1.1 ± 0.76	0.78

As regards measures of disease activity pre- and post-pregnancy between P-early and P-late patients, we did not observe any significant difference in terms of MRI activity (*p* = 0.72), ARR (*p* = 0.58) and EDSS (*p* = 0.35).

### Disease outcome comparison between patients with or without breastfeeding

Sixteen patients breastfed after delivery for a mean of 4.2 months (sd 2.4); comparing women who breastfed with those who did not, there were no differences between pre- and post-pregnancy ARR (*p* = 0.34), number of gd + lesions (*p* = 0.63) and EDSS pre- and post-cladribine (*p* = 0.65).

### Safety mother data

**Fig. 3:**
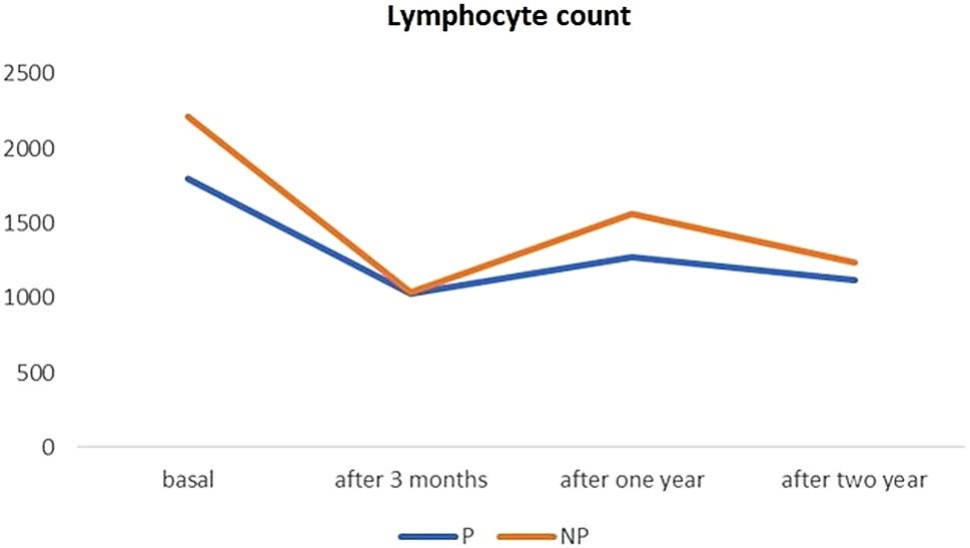
Lymphocyte kinetics after cladribine treatment in patients with (P) and without (NP) pregnancy

Within the overall cohort, we observed a reduction in the absolute lymphocytes count from 1974,5 (sd 717,70) at baseline to 1030,21 (sd 317,26) after 3 months from the first cladribrine cycle (*p* = 0.001) with a repopulation after 1 year (1399,47, sd 485) and after 2 years (1159,85, sd 265,6). As shown in Fig. 3, the trend was similar in the P and NP groups (*p* = 0.28). No adverse events were observed.

## Discussion and conclusion

After induction therapy, control of disease activity may be variable in the individual patient. To date, the only approved inductive therapies used in clinical practice are alemtuzumab and cladribine, and for both there are no studies evaluating the appropriateness of initiating other therapy either in the presence or absence of resumption of disease activity in the individual patient. For cladribine, there are expert opinions that help us to identify the patient who is a full responder, suboptimal or not responder to therapy, i.e., who has breakthrough disease after the 2nd year of therapy [9]. From clinical trials, cladribine could control disease activity for at least 4 years after the first administration with 73% relapse-free patients, and one-third of patients showing no evidence of diseaseactivity (NEDA) up to 6 years [10]. In line with this finding, real-world studies have demonstrated 64% of NEDA-3 achievement after 2 years, with the observation that this target is more achievable in naïve patients [11, 12] or switching from mild/moderate efficacy treatments [13]. This reported efficacy does not consider the possible occurrence of pregnancy, which could potentially alter treatment response by modifying MS-related inflammatory activity, due to hormonal changes occurring throughout the gestational period and immediately after childbirth [14].

In this study we investigated the effect of pregnancy on treatment response in a group of women previously treated with cladribine compared to women who did not have a pregnancy after cladribine treatment over a mean follow-up of 3.53 years from the first administration. In our study, pregnancy did not influence cladribine treatment response and there was no evidence of disease reactivation after delivery within our cohort. This finding is in line with the single-arm study of Dost-Kovalsky. et al. in which disease control was excellent both during pregnancy and the post-partum period [15]; in our cohort, we compared the measures of disease activity between patients with and without pregnancy after cladribine treatment, thus expanding the findings from the above-mentioned study. As far as we know, this is the first study that compared disease activity in terms of ARR, MRI activity and EDSS worsening between women with or without pregnancy after cladribine, and the first study to evaluate the impact of breastfeeding on disease activity, showing no evidence of disease reactivation at this stage.

Furthermore, in our cohort, the timing of pregnancy with respect to cladribine initiation (i.e., early between the first and second cycle of treatment; late after the second cycle) does not appear to influence disease activity and disability in women previously treated with cladribine; however, since we recorded a small number of pregnancy, patients should always be encouraged to complete both courses of therapy to ensure the maximum effectiveness.

In our cohort, we collected also data about two abortions that occurred between the first and second cycle of cladribine. However the small number of patients, only two, cannot lead to any conclusion, and we found no disease reactivation after miscarriage, as reported by Landi et al. [16]

Regarding newborn safety, our data are in line with reports from the registry trials and with epidemiological data on pregnancy outcomes for the general population or women with MS [17].

The lymphocytes trend does not seem to be influenced by pregnancy and no safety concern regarding mothers emerged.

This study is limited by the small sample and the retrospective study design; moreover, the lower number of patients in the NP group could be related to a bias in cladribine prescription, as it could have been selected for women willing to get pregnant. Indeed, it is well known that cladribine efficacy extends beyond year 2, therefore protecting women while attempting to conceive, without drug exposure for the fetus. Bearing these caveats in mind, we can conclude that pregnancy does not influence the effectiveness of cladribine treatment and that, in turn, appears to be protective against any postpartum reactivation. Additional studies with larger sample sizes and longer follow-up periods are needed to confirm these findings and to identify the best time frame to conceive after cladribine.

## Data Availability

The data that support the findings of this study are available from the corresponding author upon reasonable request.
